# Postprocedural radial artery occlusion rate using a sheathless guiding catheter for left ventricular endomyocardial biopsy performed by transradial approach

**DOI:** 10.1186/s12872-016-0432-y

**Published:** 2016-12-08

**Authors:** Behrouz Kherad, Clemens Köhncke, Frank Spillmann, Heiner Post, Michel Noutsias, Burkert Pieske, Florian Krackhardt, Carsten Tschöpe

**Affiliations:** 1 Department of Cardiology, Universitätsmedizin Berlin, Campus Virchow Klinikum (CVK), Berlin, Germany; 2 Privatpraxis Dr. Kherad, Große Hamburger Straße 5-11, 10115 Berlin, Germany; 3 Department of Internal Medicine I, Division of Cardiology, Pneumology, Angiology and Intensive Medical Care, University Hospital Jena, Friedrich-Schiller-University Jena, Jena, Germany; 4 German Heart Center (Deutsches Herzzentrum Berlin, (DHZB)), Berlin, Germany; 5 Berlin Center for Regenerative Therapies (BCRT), Campus Virchow Klinikum (CVK), Berlin, Germany; 6 Deutsches Zentrum für Herz Kreislaufforschung (DZHK) – Standort Berlin/Charité, Campus Rudolf Virchow, Augustenburger Platz 1, 13353 Berlin, Germany

**Keywords:** Endomyocardial biopsy, Transradial approach, Sheathless guiding catheter

## Abstract

**Background:**

For coronary interventions the arterial access via the radial artery is associated with fewer vascular access site complications, and has been shown to reduce major bleeding when compared to the femoral approach. But the endomyocardial biopsy (EMB) approach is usually done by a transfemoral or cervical access known to be associated with an increased risk of artery puncture and its potential complications (i.e., false aneurysm, artery-venous fistula) and needs post-procedural immobilization. A transradial approach for EMBs is not standardized. The aim of our study is to validate safety and efficacy of the transradial access approach for left ventricular EMB, and to define patients eligible for a safe and successful procedure.

**Methods and Results:**

We evaluated the transradial access using a 7.5 F sheathless multipurpose guiding catheter to obtain EMBs from the left ventricle (LV). 18 patients were included. The transradial success rate was 100% (18/18). There were no periprocedural cardiac complications. Immediate post-procedural ambulation could be achieved in all patients. Although radial artery pulse was confirmed by ultrasonic vascular Doppler after removal of the guide in 100% (18/18) of the patients, 50% (9/18) of the patients showed occlusion of the radial artery RAO) by duplex sonography proximal to the access site. 33% (3/9) of the patients in the RAO group and 11,1% (1/9) of the patients in the patent radial artery (RAP) group, respectively, experienced mild pain after the procedure in the right lower arm. Colour Doppler ultrasonography of the right radial artery performed 24 h after the procedure revealed radial occlusion in 50% (9/18) of the patients. The diameter of the radial artery was significantly smaller in the RAO group (*p* = 0,034), peak systolic velocity (PSV) of the right ulnar artery was significantly higher in the RAO group (*p* = 0.012). Peak systolic velocity of the opposite radial artery was significantly lower in the RAO group (*p* = 0,045). Gender, sex, diabetes, radial artery inner diameter ≤2.5 mm and lower peak systolic velocity of < 50 cm/s are predictors of RAO.

**Conclusion:**

The present study demonstrates the safety and efficacy of a transradial access for EMB using a highly hydrophilic sheathless guiding catheter.

## Background

Endomyocardial biopsy (EMB) is the technique of choice in the diagnosis of adults presenting with cardiomyopathy of unknown origin. The toxic, infectious-inflammatory, infiltrative or autoimmune processes that cause cardiac dysfunction occur at a cellular level and no other diagnostic techniques can establish the nature of the etiological agent. As well as detection of inflammation or viral genomes in the acute phase of myocarditis, EMB adds important prognostic information, which may translate to decisive therapeutic decisions [1, 2].

The 2007 American Heart Association/American College of Cardiology Foundation/European Society of Cardiology scientific statement on EMB limited its class I recommendations to unexplained new-onset heart failure of less than 2 weeks duration associated with hemodynamic compromise or unexplained new onset heart failure of 2 weeks to 3 months duration associated with a dilated left ventricle and new ventricular arrhythmias or conduction disturbances [3]. However, in a recent position statement from the ESC [1], the recommendation for EMB was extended, including patients with a pseudo-infarct presentation after excluding coronary artery disease, myocarditis and inflammatory cardiomyopathies, as well as for patients with rapidly advancing cardiomyopathy refractory to conventional therapy. This change responds to the more widespread availability of immunohistochemical and viral genome detection techniques, which improve the ability to detect the underlying cause of myocarditis. Hence, an increasing number of patients can benefit from specific treatment.

Moreover, EMBs are routinely performed in daily practice for post heart transplant patients most commonly in centres with post heart transplant patients.

Furthermore, the choice of the ventricular site for EMB is still under debate. Whereas some observe that diagnostic yield of left ventricle (LV) EMB is superior to right ventricular (RV) EMB when routine immunohistochemistry and viral genome amplification are used in suspected LV myocarditis, more recent data state that both procedures are similar when assessing inflammation or viral genome in the myocardium. However, we recently showed morphological changes such as interstitial fibrosis and cardiac collagen type I expression were more reliably found in LV EMB [4].

RV-EMB approached by right internal jugular or femoral vein and LV-EMB approached by the left or right femoral artery are commonly used, and when performed by experienced operators, both right and left EMB have very low complication rates. In a single center study that analyzed 3,048 EMB in a non-transplant setting, risk of major complications including cardiac tamponade and AV block requiring permanent pacemaker implantation was 0,12% [5, 6]. Chimenti et al. documented that over a 28-year period and over 4,000 EMB procedures performed complications appeared in only 0.33% of patients who underwent LV-EMB [7].

When LV-EMB is performed, the femoral artery is usually the access site [3, 8]. The transfemoral site, however, is associated with the risk of artery puncture and its potential complications (i.e., false aneurysm, artery-venous fistula) at the access site and limited due to post-procedural immobilization. The radial artery is increasingly used for diagnostic coronary angiography and percutaneous coronary interventions and recently recommended by the to ESC guidelines for NSTEMI [9]. Lower direct costs, fewer vascular complications, better patient acceptance and earlier ambulation are some of the direct benefits from using radial access [10]. When compared to the femoral approach, less vascular access site complications and reduced major bleeding have been reported [11–14].

Access-site vascular complications in patients undergoing transradial coronary procedures are rare but may have relevant clinical consequences [15]. Complications include spasm, occlusion or perforation of radial artery (RA), hematoma, pseudoaneurysm, arteriovenous fistula and nerve injury [16].

The purpose of this study was to analyze the safety and efficacy of a transradial approach using a sheathless guiding catheter for left ventricular EMB. In addition, we aimed to define patients eligible for such an approach, and to potentially identify patient profiles for whom the radial LV EMB approach might impose higher periprocedural risks.

## Methods

### Patient population

From January 2015 until July 2015 all patients presenting to our cath lab for EMB work-up of myocardial disease were evaluated for EMB via transradial access. 18/18 displayed a normal Allen test and gave consent for transradial EMB. Further demographic and clinical data on the patient population is given in Table 1.

**Table 1:** Patient characteristics

Patient characteristics	Occluded artery	Patent artery	*T* Test
Number of patients (n)	9	9	
Normal Allen’s test (%)	100	100	
Gender (% female)	44	22	
Age (years)	44.9 (29; 63)	47 (25-73)	0.74
Height (cm)	169 (160; 187)	176 (150;193)	0.19
Weight (kg)	77.7 (60;102)	81 (55;113)	0.68
BMI (kg/m^2^)	27 (21;36)	25 (18;32)	0.56
Creatinine (mg/dl)	0.93 (0.6;1.84)	0.93 (0.67; 1.21)	0.99
Thrombocytes (per nl)	223 (173; 296)	219 (174; 276)	0.83
INR	1.06 (0.92;1.56)	1.05 (0.94; 1.34)	0.90
LVEDP (mmHg)	20 (11;37)	22 (8; 39)	0.692
LVEDD (mm)	52 (38;61)	63 (42; 83)	0.038
LVEF (%)	39 (15; 60)	34 (15; 60)	0.548

Data are presented as mean with data range in brackets

*INR* International normalized ratio; *LVEDP* left ventricular end-diastolic pressure; *LVEDD* left ventricular end-diastolic diameter; *LVEF* left ventricular ejection fraction

### Transradial left ventricular EMB

**Fig. 1:**
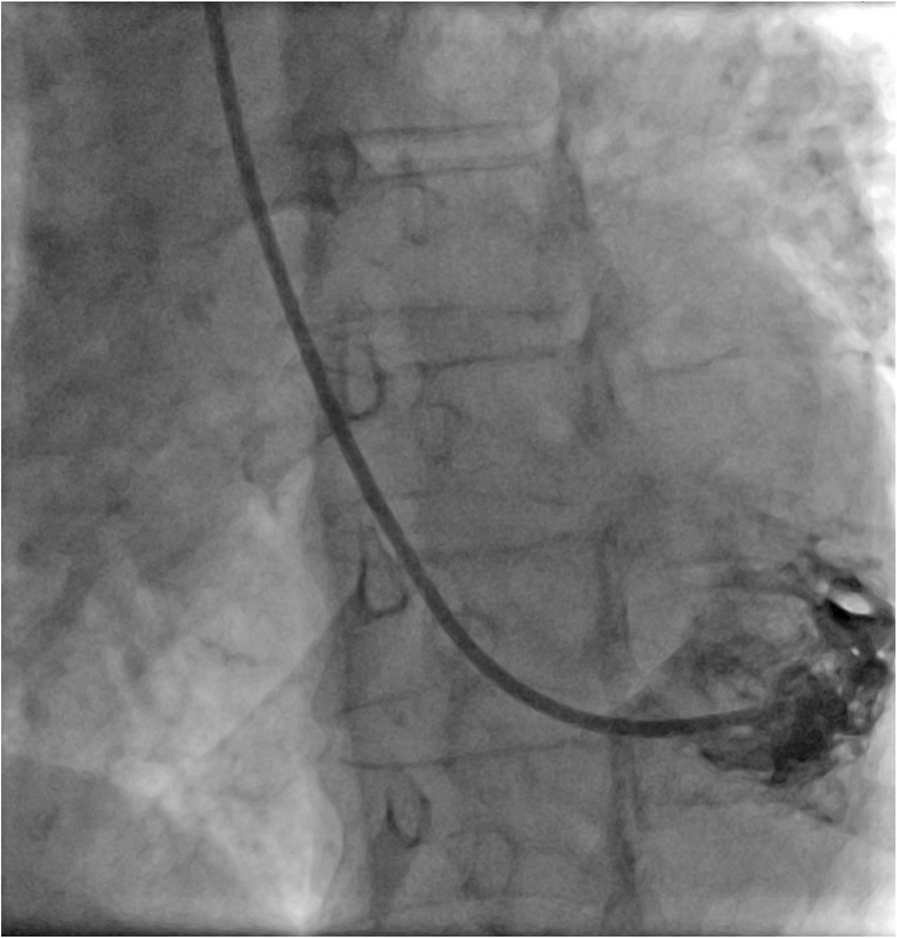
LV angiography Fluoroscopy in LAO 20° view with injection of 6 ml contrast agent to visualize the position of the 7.5 F MP-1 guiding catheter inside the left ventricle (tip pointing to the lateral LV wall)

Following local anesthesia, a 6 F sheath (Radifocus Introducer II, 10 cm, Terumo, Japan) was introduced into the right radial artery. Upon sheath introduction every patient received 3000 IU unfractionated heparin and 5 mg verapamil i.a. A 5 F pigtail catheter (Boston Scientific, USA) was now advanced into the LV. A long J-wire (260 cm, 0.03500) was advanced over the pigtail catheter to hold the ventricular position, the pigtail and the 6 F radial sheath were removed, and a 7.5 F sheathless multipurpose guiding catheter (MP1.0, Asahi Intecc, Japan) was introduced, whereas the dilatator was removed as soon as the sheathless guiding catheter reached the ascending aorta. Following removal of the dilatator the guiding catheter was carefully advanced over the wire into the LV cavity. The J-wire was now removed and a Y-connector (Copilot, Abbott Vascular, USA) was connected. The correct position of the guiding catheter tip was checked in left anterior oblique projection (LAO) 20° projection with the tip of the catheter pointing to the lateral LV wall. Once the positioning of the catheter was confirmed 6 ml of contrast agent were injected to visualize the distance of the tip to the lateral LV wall (see Fig. 1). Prior to the biopsy, activated clotting time (ACT) was checked and adjusted accordingly (ACT Aim: 250–300 s). Then, a biopsy forceps (B-18110; 1100 mm, 1.8 mm, Medizintechnik Meiners, Germany) compatible with the 7.5 french sheathless guiding catheter was inserted into the MP1.0 guiding catheter via the Y connector. The forceps were advanced under fluoroscopy close to the tip of the guiding catheter. The forceps were opened inside the guiding catheter and carefully advanced toward the lateral left ventricular wall. As soon as resistance was fluoroscopically seen, the jaws were closed and the forceps immediately retracted into the guiding catheter. Upon completion of the procedure, the sheathless guiding catheter was removed and a vascular closure device (TR Band, Terumo, Japan) was applied for hemostasis using the “patent hemostasis” protocol [17]. Briefly, the sheath was pulled out 4–5 cm and the TR Band was placed around the forearm at the site of entry. The needle cap and gauze composite was placed over the site of entry. A pulse oximeter sensor was placed over the index finger, the TR Band was tightened, and the sheath was removed. Ipsilateral ulnar artery was occluded and the hemoband was loosened till plethysmographic signal returned (confirming radial artery patency) or bleeding occurred. If radial artery patency could be maintained and hemostasis was achieved, the bands were left in place for 2 h. The patency of the radial artery was checked at least once every hour.

### Doppler ultrasonography of the radial arteries

Colour and pulsed Doppler ultrasound studies were performed by an experienced sonographer within 24 h after the procedure in all 18 patients. Radial, ulnar and brachial arteries of the access and the opposite forearm were performed using a Philips iU22 ultrasonography system (Philips Healthcare, Amsterdam, Netherlands) featuring a L9-3 MHz linear transducer.

The clinical investigations involved in this study, including the obtainment of endomyocardial biopsies, were part of the routine clinical workup of these patients. All patients gave written informed consent to the invasive clinical investigations. The use of the data for scientific evaluation was approved by the local ethics committee at the Charité–Universitätsmedizin Berlin, and all patients gave informed consent for participating in this framework of the Sonderforschungsbereich Transregio/SFB-TR 19 “Inflammatory cardiomyopathy”.

### Data analysis and statistics

Safety and efficacy parameters were: (1) procedural success, (2) quality of biopsy samples as assessed by participating pathologists, (3) radiation exposure, (4) access site vessel patency, and (5) relevant access site complications (defined as: false aneurysm, AV-fistula, drop in hemoglobin of more than two points without pericardial effusion or other overt bleeding requiring action, indication for bed rest due to the procedure). (6) Doppler ultrasonography of the radial and ulnar arteries.

Absolute numbers and percentages were computed to describe the patient population. Medians (with quartiles) or means (with standard deviation) were computed as appropriate. A comparison of qualitative variables between the groups was performed using the *χ*2 test. All statistical analyses were performed using the SAS statistical package, version 9.2 (SAS, Cary, North Carolina).

## Results

### Patient population

Mean age in both groups was similar (44.9 years in the occluded radial artery group (RAO) versus 47 years in the patent radial artery group (RAP)), whereas there were twice as many female patients in the occluded radial artery group (RAO 44% versus RAP 22%). Both groups had increased mean BMI (BMI in the RAO group was 27 kg/m^2^ versus 25 kg/m^2^ in the RAP group). Both groups displayed severely impaired LV-EF function (mean LVEF in the RAO group 39% versus 34% in the RAP group), whereas the left ventricular end-diastolic diameter was significantly larger in the RAP group (LVEDD in the RAO group 52 mm versus 63 mm in the RAP group, *P* < 0,05)). The blood results were similar in both groups. Additional patient characteristics can be viewed in Table 1.

### Procedural characteristics, safety and efficacy

Depending on the clinical indication, the transradial invasive evaluation encompassed the whole spectrum from EMB as a standalone procedure or combined left/right heart catheterization. There was no need to cross over to femoral access. Thus, the success rate of EMB via transradial access was 100% (18 of 18) in both groups. Table 2 provides a detailed overview of the procedures performed.

**Table 2:** Procedural details

Procedural details	Occluded artery	Patent artery	*T* Test
Simultaneous coronary angiogram (%)	44.44	44.44	
ACT (sec)	241	278	0.469
Total fluroscopy time (Min)	8. 24 (5.41; 12.35)	6.47 (3.77;9.57)	0.098
Dose area product (cGy cm2)	1314.12 (213; 4366)	1475 (243;3433)	0.800

Data are presented as mean with data range in brackets

The mean duration of the invasive evaluation (RAO 8,24 min versus 6,24 min in the RAP group; *P* > 0,05) and the mean dose area product (RAO 1314,12 cGy cm^2^ versus 1475 cGy cm^2^ in the RAP group; *P* > 0,05) were similar in both groups (Table 2). The number of biopsy samples harvested were 10 samples per patient in order to optimize diagnostic accuracy and reduce sampling errors [18]. All biopsy samples harvested via transradial access where graded as good or excellent quality by the pathologists involved.

### Procedural success/complications

By using the transradial approach, we were able to obtain left ventricular EMBs in all 18 patients. No intraprocedural complications were encountered in both groups, in particular no cases of pericardial effusion, thromboembolism or refractory radial artery spasm. Immediate post-procedural ambulation could be achieved in all patients as for no patient any bed rest was assumed necessary.

### Postprocedural complications

All patients had radial artery pulse confirmed by ultrasonic vascular Doppler 24 h after removal of the guide.

33% (3/9) of the patients developed mild right lower arm pain in the RAO Group, whereas 1 Patient (11.11%) developed mild lower arm pain in the RAP group. No patient developed pain requiring further intervention.

### Doppler ultrasonography of the radial and ulnar arteries

All patients (18/18) underwent Colour doppler ultrasonography of the radial and ulnar arteries within 24 h after the procedure was performed (Table 3).

**Table 3:** Doppler ultrasonography of the radial arteries

Doppler ultrasonography	Occluded artery	Patent artery	*T* Test
Diameter right radial artery (mm)	2.52 (2;2.8)*	2.86 (2.5;3.8)	0.034
Flow Profile (triphasic in %)	0	100	
PSV (cm/s)	*	38.1 (10;96.3)	
Flow Volume (ml/min)	*	17.93 (4.5; 59.6)	
Diameter right distal ulnar artery (mm)	2.62 (2.1; 3.6)	2.87 (2.3;3.47)	0.182
Flow Profile (triphasic in %)	100	100	
PSV (cm/s)	85.2 (60.8;154)	57.2 (36.3; 90)	0.012
Flow Volume (ml/min)	44.72 (18.3;68.3)	47 (10.3; 176)	0.452
Diameter left radial artery (mm)	2.51 (2.2;3)	2. 72 (2.4; 3.3)	0.061
Flow Profile (triphasic in %)	100	100	
PSV (cm/s)	49.15 (35; 73.2)	65.4 (35; 88)	0.045
Flow Volume (ml/min)	23.87 (12; 46.6)	34.97 (9.5; 128)	0.196
Diameter left distal ulnar artery (mm)	2.53 (2.2; 3.2)	2.81 (2.3; 3.6)	0.141
Flow Profile (triphasic in %)	100	100	
PSV (cm/s)	55.5 (38;70.3)	50. 31 (25.2; 85)	0.248
Flow Volume (ml/min)	30.8 (9.6;55.7)	35. 88 (10.1; 128)	0.364

*PSV* Peak systolic velocity

Doppler ultrasonography of the right radial artery revealed radial occlusion in 50% (9/18) of the patients. The diameter of the radial artery was significantly smaller in the RAO group (Diameter right radial artery (mm) RAO: 2.52 vs. RAP: 2.86 mm; *p* = 0.034).

Doppler ultrasonography of the right ulnar artery showed no significant difference between the two groups in size (Diameter right ulnar artery (mm) RAO: 2,62 vs. RAP 2,87 mm; *p* = 0,182). However, the peak systolic velocity (PSV) was significantly higher in the RAO group (PSV (cm/s): RAO 85.2 vs. RAP: 57.2, *p* = 0.012).

On the contralateral site the diameter of the left radial artery was smaller in the RAO group (Diameter left radial artery (mm) RAO: 2.51 vs. RAP: 2.72 mm; *p* = 0,061). Peak systolic velocity (PSV) was significantly lower in the RAO group (PSV (cm/s): RAO 49.15 vs. RAP: 60.3, *p* = 0.045).

Left ulnar artery showed no significant difference between the two groups in size (Diameter left ulnar artery (mm) RAO: 2.53 vs. RAP 2.81 mm; *p* = 0.14). or PSV (PSV (cm/s): RAO 55.5 vs. RAP: 50.31, *p* = 0.248).

## Discussion

The results of our study showed that EMB using a transradial approach is feasible and efficient.

By using the transradial approach, we were able to obtain left ventricular EMBs in all 18 patients without the need to convert to transfemoral access. No intraprocedural complications were encountered, in particular no cases of pericardial effusion, thromboembolism or refractory radial artery spasm. All obtained EMB samples were graded as excellent quality by the pathologist. These results are similar to two recently published series of transradial approach for LV-EMB [19, 20].

**Fig. 2:**
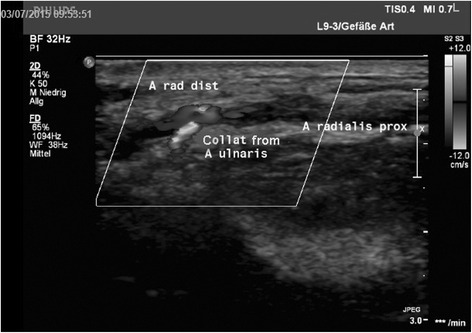
Color coded Doppler sonography shows origin of the ‚palpabel pulse’ in the distal radial artery (A rad dist): Due to a collateral from the ulnar artery (Collat from A ulnaris) a pulse was palpabel in the distal radial artery even though the proximal radial artery (A radialis prox) was occluded

However, our study also showed that a subclinical radial artery occlusion (RAO) occurs in 50% of patients after transradial endomyocardial biopsies. On clinical examination, most of the patients with color-coded Doppler sonography documented RAO (6/9) still had a ‘palpable radial pulse’. This palpable pulse however was not a radial but an ulnar pulse fed via the palmar arch (see Fig. 2).

The incidence of RAO in our study was higher than in previous studies. This may be due to the fact that in contrast to previous studies we are among one other study, the only study with ultrasonic vascular Doppler based diagnosis of RAO [21]. Although, we were able to detect a palpable radial pulse in most of the patients with documented RAO, using ultrasonic vascular Doppler based diagnosis we unmasked a RAO rate of 50%. It has been shown that patients with RAO can have palpable radial pulse even with occluded proximal segment of the radial artery because of collateral circulation from the palmar arch [17, 22]. The incidence of RAO can be detected by vascular Doppler ultrasound only, studies using RAO ultrasonic vascular Doppler based diagnosis of RAO, showed that the RAO rate was well correlated with the size of transradial sheath (13.7 vs 30.5% in 5 Fr vs 6 Fr sheath) [23].

Secondly, although a French study on adult patients with suspected coronary artery disease reported a radial artery diameter to be 3.7 ± 0.8 mm [24] we observed much smaller radial artery diameter of 2.7 ± 0.4 mm (range: 2.0-3.8 mm). These observed radial diameters are more similar to diameters observed in Asian populations [25].

Thirdly patient in both groups were young and the percentage of female patients was high in both groups, whereas there were twice as many female patients in the RAO Group.

Recently, a univariate analysis of predictors of RAO including patients who underwent diagnostic angiography and angioplasty with a 5 French and 6 French sheath showed that the presence of younger age and female sex were again strong predictors of post-procedural RAO. The mean age of that study was significantly higher than our mean age (Mean age: 5 F (65.1 ± 10.8) and 6 (64.9 ± 11.0)) and the percentage of females was similar than in our study (female patients 5 F 97 (36.6%) and 6 F 186 (38.4%) [17]. Another predictor of RAO in a prospective study of RAO was diabetes mellitus [23].

In our study, a total of 3 patients had a radial artery diameter less than the radial sheath diameter used for TR-EMB (7.5 Fr with an outer diameter of 2.49 mm). All of these 3 patients developed RAO. Therefore, in our study, the occlusion rate was 100% in patients with radial artery to sheath ratio ≤1 as compared to 40% if the ratio was >1.

The artery to sheath ratio seems to be a contributing factor for RAO since Saito et al. showed that a radial artery diameter/sheath-diameter ratio <1 is associated with a reduction in distal flow [26]. Interestingly even in the RAP group the post procedural PSV was low in the radial artery (PSV 38,1 cm/s (10; 96,3)). However, Garg et al. showed analyzing patients undergoing coronary interventions using a 6 French guiding catheter that radial artery to sheath ratio ≤1 was not an independent predictor for RAO on multivariate analysis [25].

## Conclusion

In conclusion, the present report shows the feasibility of using a sheathless catheter to guide access for LV-EMB. However, duplex sonography revealed a high number of post procedural asymptomatic radial occlusions. Gender, age, Diabetes mellitus, radial artery inner diameter ≤2.5 mm and lower radial peak systolic velocity of ≤ 50 cm/s are likely predictors of RAO.
